# Temperature-Driven Transformation of the Crystal and Magnetic Structures of BiFe_0.7_Mn_0.3_O_3_ Ceramics

**DOI:** 10.3390/nano12162813

**Published:** 2022-08-16

**Authors:** Dmitry V. Karpinsky, Maxim V. Silibin, Siarhei I. Latushka, Dmitry V. Zhaludkevich, Vadim V. Sikolenko, Roman Svetogorov, M. I. Sayyed, Nouf Almousa, Alex Trukhanov, Sergei Trukhanov, Alexei А. Belik

**Affiliations:** 1 Scientific-Practical Materials Research Centre of NAS of Belarus, 220072 Minsk, Belarus; 2 Institute for Advanced Materials and Technologies, National Research University of Electronic Technology “MIET”, 124498 Zelenograd, Moscow, Russia; 3 Joint Institute for Nuclear Research, 141980 Dubna, Russia; 4 NRC “Kurchatov Institute”, Acad. Kurchatov Sq. 1, 123182 Moscow, Russia; 5 Department of Physics, Faculty of Science, Isra University, Amman 1162, Jordan; 6 Department of Physics, College of Science, Princess Nourah bint Abdulrahman University, P.O. Box 84428, Riyadh 11671, Saudi Arabia; 7 Smart Sensors Laboratory, National University of Science and Technology MISiS, 119049 Moscow, Russia; 8 International Center for Materials Nanoarchitectonics (WPI-MANA), National Institute for Materials Science (NIMS), Namiki 1-1, Tsukuba 305-0044, Ibaraki, Japan

**Keywords:** crystal structure, magnetic properties, phase transitions, synchrotron and neutron diffraction, functional magnetic oxides

## Abstract

The compound BiFe_0.7_Mn_0.3_O_3_ consisting at room temperature of coexistent anti-polar orthorhombic and polar rhombohedral phases has a metastable structural state, which has been studied by laboratory X-ray, synchrotron and neutron diffraction, magnetometry, differential thermal analysis, and differential scanning calorimetry. Thermal annealing of the sample at temperatures above the temperature-driven phase transition into the single phase rhombohedral structure (~700 K) causes an increase of the volume fraction of the rhombohedral phase at room temperature from ~10% up to ~30%, which is accompanied by the modification of the magnetic state, leading to strengthening of a ferromagnetic component. A strong external magnetic field (~5 T) applied to the sample notably changes its magnetic properties, as well as provides a reinforcement of the ferromagnetic component, thus leading to an interaction between two magnetic subsystems formed by the antiferromagnetic matrix with non-collinear alignment of magnetic moments and the nanoscale ferromagnetic clusters coexisting within it. The modification of the structural state and magnetic properties of the compounds and a correlation between different structural and magnetic phases are discussed focusing on the effect of thermal annealing and the impact of an external magnetic field.

## 1. Introduction

During the last decades, the complex oxides of transition metals having multiferroic properties have drawn a particular attention of researchers [1,2,3]. It is known that the structural state of such compounds can be notably changed by chemical doping, and a change in stoichiometry also allows a modification of their properties [4,5,6,7]. Moreover, the compounds with chemical compositions within the morphotropic phase boundary are characterized by enhanced susceptibility to external stimuli such as electric and magnetic field, temperature, and pressure, thus enhancing the area of their practical applications including sensors of electric and magnetic fields, actuators, and other electrical devices [8,9,10].

Bismuth ferrite (BiFeO_3_) is one of the widely known single-phase multiferroics with ferroelectric and magnetic transitions occurred at high temperatures, viz. 1100 K and 650 K, respectively [11,12]. While pristine BiFeO_3_ is characterized by severe drawbacks critical for technological applications, i.e., high leakage current, low remnant magnetization, and so on, chemical doping can be utilized as an effective tool to improve its functional properties [13,14,15]. Chemical substitution of Bi ions by alkali-earth ions or rare-earth elements and/or iron ions by other transition metal ions can significantly modify the structure and to improve the electrical and magnetic properties of these complex oxides [16,17,18,19,20,21]. In particular, the substitution of Fe ions by Mn ions leads to the structural phase transition from the rhombohedral structure (space group *R3c*) to the orthorhombic structure (s.g. *Pnma*), and then to the structure with monoclinic distortion (s.g. *C2c*) [17,22]. Upon the mentioned chemical doping, the magnetic structure of the compounds transforms from the spatially modulated spin structure to the antiferromagnetic one with non-collinear alignment of magnetic moments, and then to the ferromagnetic structure governed by the orbital order of Mn^3+^ ions [17,22,23,24].

It is known that the compounds BiFe_1-x_Mn_x_O_3_ with chemical compositions in the vicinity of the morphotropic phase boundaries, viz. the rhombohedral–orthorhombic phase boundary (x~0.3) and the orthorhombic–monoclinic boundary (x~0.6), are characterized by metastable structural and magnetic states [17,22]. In particular, the crystal structure of the solid solutions with x ≈ 0.3 and x ≈ 0.6 exhibit an irreversible structural transition after annealing at T~700 K, which causes an increase of the volume fraction of the structural phase specific for a high temperature range, viz. the rhombohedral and the orthorhombic phases, respectively [17]. The magnetic phase of the solid solutions with chemical compositions in the morphotropic phase boundaries can also be notably changed by thermal annealing or by applying an external magnetic field [20,25], while the origin of the modification of the magnetic phases occurring under external stimuli is still unclear and the correlation between the respective structural and magnetic phases is questionable [17,20,26,27].

In the present study, we have focused our investigations on the structure and magnetic properties of BiFe_0.7_Mn_0.3_O_3_ ceramics, which are characterized by the two-phase structure at T ≈ 300 K and consist of the dominant antipolar orthorhombic phase and the minor rhombohedral phase. A combination of the results obtained by the diffraction methods (including X-ray and neutron radiation) and magnetometry measurements has allowed a determination of the variations occurring in the magnetic sublattice of the compound subjected to external magnetic field and thermal annealing, as well as a correlation between the mentioned changes, and the modification of the crystal structure is also discussed.

## 2. Experimental

BiFe_0.7_Mn_0.3_O_3_ ceramic compounds were prepared from stoichiometric mixtures of the simple oxides Bi_2_O_3_, Fe_2_O_3_, and Mn_2_O_3_ using a high-pressure, high-temperature method at 6 GPa and 1400 K, respectively, annealed for 90 min in Au capsules [28]. Note that the synthesis of BiFe_0.7_Mn_0.3_O_3_ ceramics at an ambient pressure using a conventional ceramic method produces a different crystal structure modification [28,29]. The mentioned synthesis conditions allowed a preparing of ceramic compound with an average size of the crystallites of about several hundred nanometers. The crystal structure of the solid solutions was analyzed based on the diffraction measurements performed by laboratory diffractometer X’pert Pro (Malvern Panalytical Ltd., Kraków, Poland) and synchrotron powder diffraction (SPD) facilities (KMC-2, BESSY-II [30], BL02B2 at SPring-8, and XSA beamline of Kurchatov Synchrotron Radiation Source [31]). Neutron diffraction experiments were performed using the HRPT diffractometer (PSI) [32]. The diffraction information were refined by FullProf software (Rietveld method, ver. 0.7.2017, Grenoble, France) [33]. Magnetization measurements were performed using Magnetic Properties Measurement System (Cryogenic Ltd., London, UK). Differential thermal analysis (DTA) and differential scanning calorimetry measurements (DSC) were performed using Netzsch Phoenix setup (Selb, Germany) (nitrogen was used as a medium gas) in the range of 300–700 K.

## 3. Results

**Figure 1:**
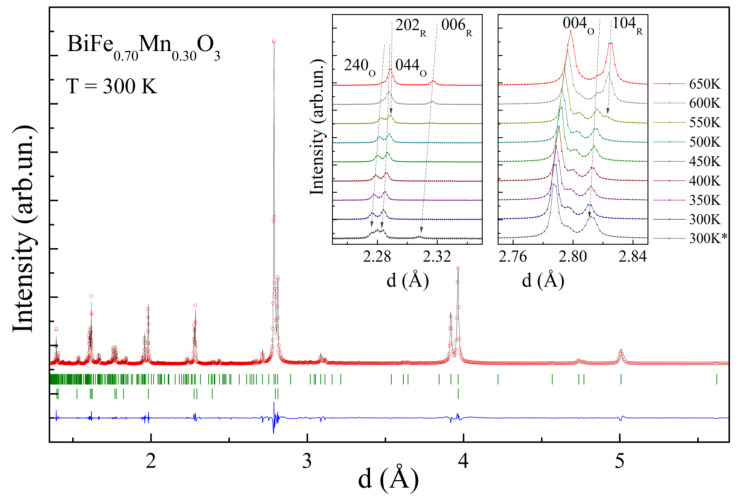
Synchrotron diffraction pattern recorded for compound BiFe_0.7_Mn_0.3_O_3_ at room temperature and calculated using the two-phase model (upper ticks—the orthorhombic phase). The insets show temperature changes of the reflections ascribed to the *R*- and *O*-phases (the pattern marked as 300 K* denotes the data obtained for the annealed sample).

**Figure 2:**
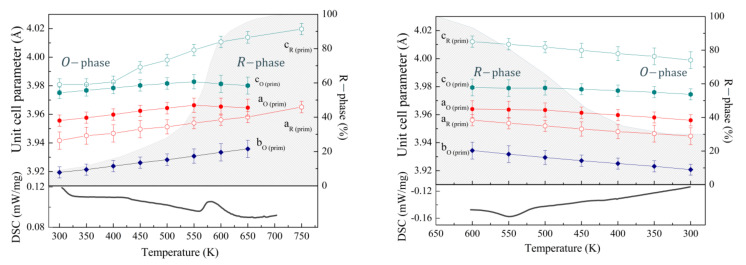
The unit cell parameters of the *O*- and *R-*phases at different temperaturs based on the SPD and NPD data on heating (**left**) and cooling (**right**) cycles; the estimated volume ratio of the phases is denoted by the dashed areas; the DSC curves are depicted at the bottom of the images.

According to the previous investigations, an increase in the Mn ions’ concentration in the compounds BiFe_1−x_Mn_x_O_3_ leads to the change in crystal structure from polar active rhombohedral to antipolar orthorhombic, followed by the polar monoclinic structure. The phase transformations are accompanied by a creation of the two-phase structural regions consisting of stable neighboring structural phases in the dopant concentration ranges with x ≈ 0.3 and x ≈ 0.6, respectively [25]. At temperature T ≈ 300 K, the as-synthesized compound with x ≈ 0.3 is characterized by a coexistence of the dominant antipolar orthorhombic phase with volume fraction ~90% (s.g. *Pnma*, metric √2a_p_∙4a_p_∙2√2a_p_, a_p_-primitive perovskite unit cell parameter) and the minor rhombohedral phase (s.g. *R3c*, √2a_p_∙√2a_p_∙2√3a_p_) (Figure 1). A variation in temperature notably changes the ratio of the coexisting structural phases. Thus, at a low temperature (T ≈ 5 K), the amount of the rhombohedral phase is neglegible according to the diffraction data and the crystal structute of the compound becomes orthorhombic with single phase strucuture. An analysis of the diffraction patterns recorded at different temperatures has allowed to determine the unit cell parameters ascribed to both phases as a function of temperature (Figure 2).

An analysis of the temperature-dependent diffraction spectra indicates a prominent increase of the reflections specific for the rhombohedral phase, which points at an increase of the volume fraction of this phase (Figure 1). Heating of the sample leads to a steady growth of the unit cell parameters, while the amount of the rhombohedral phase only slightly increases and reaches ~20% at a temperature of 450 K (Figure 2). At temperartures above 500 K, the amount of the rhombohedral phase increases more rapidly, thus reaching ~50% at T~550 K, which is accompained by notable changes in the parameters specific for both structural phases. Thus, while the *a-* and *c-*parameters of the orthorhombic phase notably decrease, the *b-*parameter maintains gradual growth in the temperature range of 500–650 K. The stated changes of the unit cell parameters point at a reduction in the antipolar distortion of the lattice and provide a stabilization of a more symmetrical structure. After a nearly stable value of the *c-*parameter of the rhombohedral phase at temperarures below 400 K, above this temperature, the parameter rapidly increases, while there is no distinct changes in the *a-* and *c-*parameters of the rhombohedral phase, and then the structural phase transition from the orhorhombic to the rhombohedral structure occurs more rapidly (Figure 2). In accordance with the DSC and DTA data, the phase transformation from the orthorhombic to the rhombohedral occurrs in the temperature range of 570–600 K (Figure 2), while the diffraction data point to the presence of the orthorhombic phase at temperatures above 650 K. An increase in temperature above 700 K leads to the formation of another phase refined as a cubic-like phase, while the rhombohedral phase remains dominant at temperature of about 750 K, above the mentioned temperature begins a chemical degradation.

Upon a temperature decrease, the parameters of the respective structural phases show dependencies showing a notably different behavior as compared with that observed during a heating cycle. The amount of the rhobohedral phase calculated at room temperature for the sample after cooling is about 30%, which is significantly larger than for the sample before heating. A stabilization of the rhobohedral phase in the compound subjected to thermal annealing at 700 K attests that the rhombohedral structure having a larger unit cell volume and higher symmetry as compared with the orthorhombic structure is more energenically favorable.

**Figure 3:**
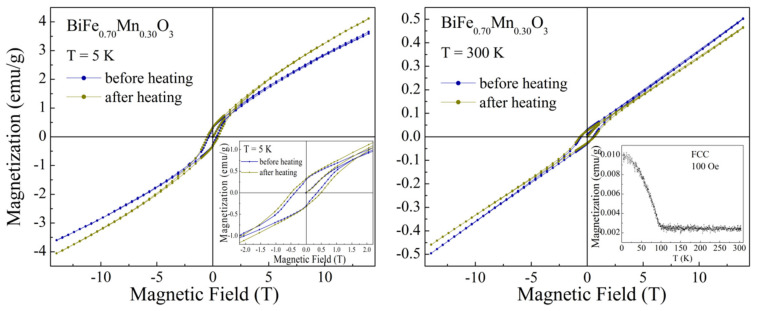
Isothermal magnetization dependencies recorded at temperatures of 5 K (**left**) and 300 K (**right**) before and after annealing at 700 K. The inset shows the FCC curve obtained in a field of 100 Oe.

The modification of the structure of the compound after heating also leads to a transformation of its magnetic properties. The sample before heating is characterized by the G-type antiferromagnetic structure, as confirmed by the magnetometry and the results of neutron diffraction measurements. The isothermal magnetization dependencies obtained for the compound at 5 K (Figure 3) testify to the presence of a weak ferromagnetic component with the resulting value of remanent magnetization of ~0.3 emu/g, which coexists with a dominant antiferromagnetic phase. The small ferromagnetic component is caused by a non-collinear alignment of the magnetic moments ascribed to Fe and Mn ions, which is caused by anti-symmetric exchange interactions [34] formed in the long-range antiferromagnetic structure. The value of remanent magnetization is similar to that observed for the BiFeO_3_ compounds, where Bi ions are substituted by the rare-earth ions, and associated with partial destruction of the spatially modulated spin structure [13,35,36]. The isothermal magnetization curves recorded for the compound annealed at 700 K are characterized by the increased value of the coercive force (~0.48 T) as compared with that determined before heating (~0.37 T) (Figure 3). A notable increase (~25%) in the value of coercivity is most probably caused by an increased amount of uncompensated magnetic moments located in the phase boundaries between the *R-* and *O-*structural phases; these phase boundary magnetic moments are coupled to the antiferromagnetic matrix because of the exchange bias effect, and thus contribute to coercivity.

It should be noted that a chemical doping of pristine BiFeO_3_ by Mn ions also causes a frustration of the negative exchange interactions between Fe^3+^ ions. Analysis of the chemical bond lengths Fe–O (Mn–O) calculated from the neutron diffraction data testifies to the formation of a small amount (less than 10%) of Mn ions in 4+ oxidation state, which can provide positive exchange interactions Mn^4+^ (3d^3^)–O-Fe^3+^ (3d^5^) and form short-range ferromagnetic clusters. The magnetic state of the compound is described assuming a coexistence of the nanoscale ferromagnetic clusters in the antiferromagnetic matrix which is in accordance with the results of magnetometry, viz. the M(T) magnetization dependencies show a magnetic transition temperature at T_C_ ~100 K (Figure 3, inset). Annealing of the sample at 700 K leads to a stabilization of the *R-*phase, and thus provides an increase in the amount of uncompensated magnetic moments in the phase boundaries, which provides a stabilization of the ferromagnetic component formed by Mn and Fe ions. A strengthening of the ferromagnetic component is observed at the isothermal magnetization data, viz. the value of remnant magnetization at T ~5 K is about 10% larger for the compound after thermal annealing.

**Figure 4:**
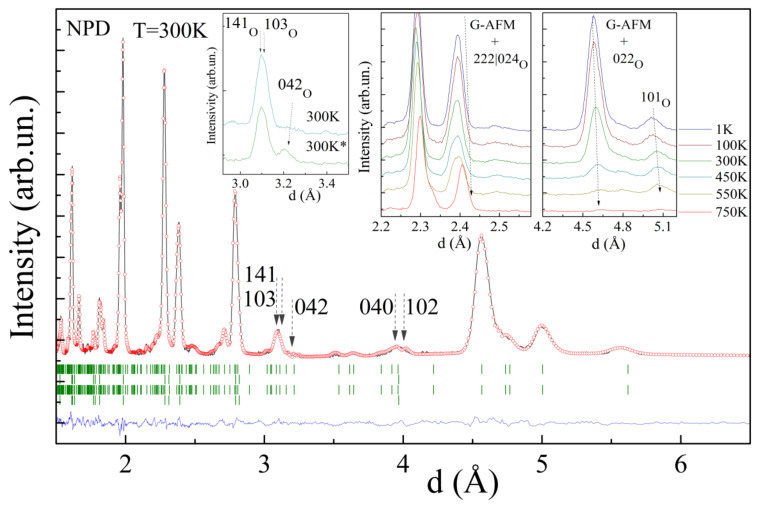
NPD pattern of the compound refined in the two-phase model (upper ticks row denotes the *O*-phase; second row denotes the *R-*phase; bottom rows denote related magnetic phases). The insets show the temperature-driven evolution of the specific reflections consisting of the components of both magnetic and nuclear scattering; the pattern marked as 300 K* denotes the data obtained for the annealed sample.

In spite of the increased value of magnetization, the ferromagnetic component remains to be of a short range order character as determined by the NPD data. Analyses of the NPD data obtained for the compound before and after thermal annealing revealed only slight changes in the magnetic structure. The magnetic structure remains dominantly G-type antiferromagnetic with the transition temperature of ~450 K (Figure 4), while careful analysis of the reflections attributed to magnetic scattering has shown some changes. In particular, the reflections ascribed to the G-type AFM structure decreases in intensity and the calculated value of the magnetic moment reduces from ~2.2 μ_B_ to ~2.0 μ_B_ at room temperature (m ≈ 4.4 μ_B_ at T = 1 K, where the spin only value for the stoichiometric compound is 4.7 μ_B_) (Figure 4, insets).

**Figure 5:**
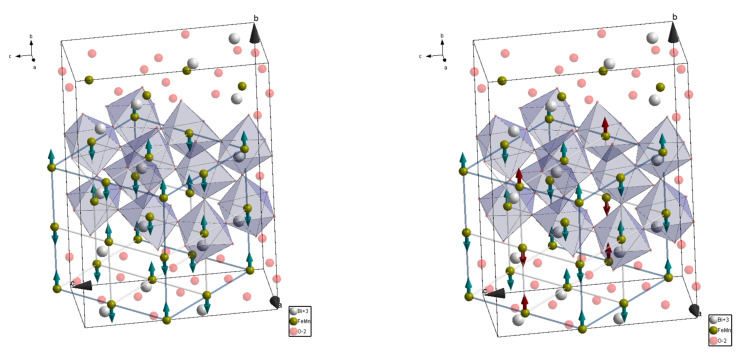
Schematic presentation of the orthorhombic lattice; the alignment of the magnetic moments are marked by the arrows, before thermal annelaing at 700 K—**left** image, and after—**right** image.

The intensity of some reflections, attributed to ferromagnetic scattering, increased slightly. It should be noted that, because of the lattice metric used to describe the structural phases, the reflections specific for the ferromagnetic scattering overlap those specific for nuclear scattering, thus hampering a precise calculation of the magnetic moments as well as the volume fraction of the phases. Meanwhile, growth in the reflections 042 and 102|040 has allowed to itemize a modification of the magnetic structure after annealing (Figure 4, insets). In particular, a modification of the intensities of the mentioned reflections can be explained assuming a reorientation of two magnetic moments of each of the eight magnetic moments attributed to the primitive perovskite cell. A modification in the orientation of the magnetic moments specific to the G-type antiferromagnetic structure occurs in the next way, viz. one of the magnetic moments oriented along the *b*-axis changes its direction to the opposite one, thus leading to a local positive coupling between the nearest ions (Figure 5). The reorientation of two magnetic moments leads to a partial frustration of the negative exchange interactions forming the G-type antiferromagnetic structure, while it does not lead to a formation of a long-range ferromagnetic phase.

While the thermal annealing of the compound only slightly changes its magnetic structure, an application of external magnetic field drastically modifies its magnetic properties. It is already recognized, that the magnetic properties of this solid solution notably depend on magnetic prehistory, and the changes in the magnetic properties were considered to have either an “extrinsic” origin, assuming chemical inhomogeneity of the sample [20], or to be produced by the modification of the volume fraction of the structural phases and associated magnetic phases [25]. The authors consider the second scenario as more plausible, as a combination of the obtained NPD and magnetization data have allowed to justify this model.

**Figure 6:**
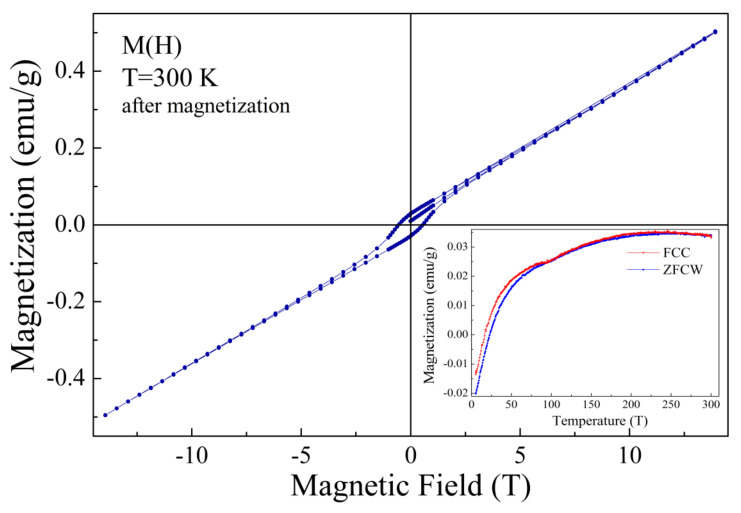
Isothermal magnetization dependence recorded at 300 K for the compound subjected to external magnetic field of 14 T. The inset shows ZFC and FC dependencies measured at 100 Oe.

A decrease in the magnetization value observed at the ZFC and FC dependencies of the compound subjected to external magnetic field of 14 T (Figure 6, inset) can be explained assuming the presence of two ferromagnetic components having different orientation and anisotropy. In particular, the ferromagnetic component formed because of the non-collinear orientation of magnetic moments of the antiferromagnetic matrix (weak ferromagnetism) from one side and the ferromagnetic clusters formed by the phase boundary magnetic moments from the other side.

These ferromagnetic components are characterized by different anisotropy and resulting magnetization values. Cooling of the pristine compound leads to an appearance of notable spontaneous magnetization below 100 K (see Figure 3, inset), which is caused by the magnetic moments at the phase boundary regions forming nanoscale clusters. Above the temperature of 100 K, the thermal agitation factor prevails that of the exchange interaction between the magnetic moments, thus leading to the magnetic phase transition associated with a destruction of the ferromagnetic coupling. The compound has been previously subjected to strong magnetic field, and a temperature decrease leads to a stabilization of the ferromagnetic component formed by the phase boundary magnetic moments with a total magnetization vector aligned in the opposite direction of the magnetization vector attributed to the weak ferromagnetism and small external magnetic field (~100 Oe) applied during the measurement procedure. A further decrease in temperature leads to a strengthening of the ferromagnetic component formed by the phase boundary magnetic moments and to a reorientation of the related magnetization vector in the direction opposite to that attributed to the weak ferromagnetism associated with the antiferromagnetic matrix, thus leading to a negative resulting magnetization value observed at temperatures below ~25 K. The magnetization reversal effect is caused by an exchange bias phenomena and strongly depends on the value of the applied external magnetic field. Based on the results of magnetization measurements and the NPD data, one can conclude that the ferromagnetic component formed by the antiferromagnetic matrix is significantly larger than that, formed by the phase boundary magnetic moments, and a compete characterization of these ferromagnetic components can be highlighted by an optimization of the magnetic annealing conditions. Thus, a lower value of external magnetic field can lead to a more pronounced magnetization reversal phenomenon, leading to a notable shift in the magnetic hysteresis loops, as confirmed by the data published in previous papers [20,37]. Similar evolution of the magnetic properties has been observed in other complex oxide systems characterized by an exchange bias between the dominant antiferromagnetic structure and minor ferromagnetic component, regardless of its origin [38,39,40].

## 4. Conclusions

The synchrotron and neutron diffraction experiments simultaneously with the magnetometry data and the DSC (DTA) results have allowed a clarification of the temperature-driven modification of the crystal and magnetic phases of BiFe_0.7_Mn_0.3_O_3_ ceramic. It is shown that heat treatment of the compound at T~700 K increases the fraction of the rhombohedral phase, which is accompanied by an increase in the coercivity and the magnetization value. The modification of the magnetic structure observed for the annealed sample is associated with an increased amount of the uncompensated magnetic moments located at the phase boundary region, which frustrate the exchange interactions in the antiferromagnetic matrix. The sample subjected to a strong external magnetic field is characterized by a compete characterization of the two ferromagnetic components formed by the non-collinear alignment of the magnetic moments of the antiferromagnetic matrix and by nanoscale ferromagnetic clusters formed by the phase boundary magnetic moments. An effect of magnetization reversal observed for the compound after magnetization is caused by an exchange bias attributed to the mentioned magnetic phases, thus leading to a negative value of the resulting magnetization. The mentioned anomaly is caused by an orientation of the magnetization vector of the ferromagnetic component formed by ferromagnetic clusters in the opposite direction to that attributed to the weak ferromagnetism associated with the antiferromagnetic matrix.

## Data Availability

Data supporting the reported results will be provided by the authors upon request.
